# The farm cost of decreasing antimicrobial use in dairy production

**DOI:** 10.1371/journal.pone.0194832

**Published:** 2018-03-22

**Authors:** Guillaume Lhermie, Loren William Tauer, Yrjo Tapio Gröhn

**Affiliations:** 1 Department of Population Medicine and Diagnostic Sciences, College of Veterinary Medicine, Cornell University, Ithaca, United States of America; 2 Dyson School of Applied Economics and Management, Cornell SC Johnson College of Business, Cornell University, Ithaca, United States of America; University of Illinois, UNITED STATES

## Abstract

Antimicrobials are used in animal agriculture to cure bacterial infectious diseases. However, antimicrobial use (AMU) inevitably leads to the selection of resistant bacteria, potentially infecting humans. As a global public threat, antimicrobial resistance has led policy makers to implement regulations supervising AMU. The objective of our research was to investigate the farm impact of several potential policies aimed at decreasing AMU. We modeled a dairy herd of 1000 cows with an average level of disease prevalence for the nine most frequent bacterial dairy diseases found in western countries. We calculated the farm net costs of AMU prohibition, as well as cost increases in antimicrobial treatments prices, and an increase in the milk withdrawal period after AMU. Sensitivity analyses were conducted to assess the impact of output and input prices, and disease prevalence. At a mean disease prevalence, the average net costs of not using antimicrobials were $61 per cow per year greater compared to a scenario modeling current farm AMU. The model predicted that the minimum and maximum increased costs associated with AMU prohibition were $46 and $73 per cow per year compared to current AMU. In each scenario, the cost difference increased with disease prevalence. Sensitivity analysis showed that the three stochastic variables which most significantly influenced the cost difference were respectively, cow replacement prices, cow slaughter price, and the milk price. Antimicrobial price increases of a factor of five, or extending the milk withdrawal period by 15 days, resulted in increasing the costs of diseases to a level where the farmer was better off not using antimicrobials. Our results suggest that the farm level costs of AMU prohibition in many cases might be minor, although the consequences of any policy instrument should be carefully evaluated to reach the ultimate goal of decreasing AMU without threatening the sustainability of milk production.

## Introduction

The use of antimicrobials (AMU) generates the negative side effect of selecting resistant bacteria. As a result the increased mortality and treatments costs associated with antimicrobial resistance (AMR) in humans have shifted AMR from a medical to a socio-economical challenge, that have led policymakers to implement measures to mitigate AMU in both human and animal health [1–3]. There is evidence that AMU in animal agriculture contributes to an alarming rise in therapeutic failures in humans, even though the quantitative contribution to this failure remains unclear [4]. This ambiguity justifies policy to decrease AMU in animal production. However, a major difference between human health and animal agriculture is that in addition to a therapeutic objective, AMU in animal production further fulfills an economic objective, as the presence of infectious diseases in the farms threatens the efficiency and profitability of the production process [5]. In dairy production, antimicrobials are used to prevent and cure infectious diseases (ID). As a result, reducing AMU may lead to increased mortality and/or morbidity for the animals affected, may increase the incidence of ID within the herd, and consequently may decrease production output entering the food chain.

The major ID afflicting animals in dairy operations depend on their age and their stage of production. In lactating cows, antimicrobial treatment (AMT) is used to prevent or cure clinical mastitis, subclinical mastitis, metritis, retained placenta, lameness and respiratory disease. These diseases represent the prominent ID observed in a herd [6–8]. Furthermore, AMU at drying-off at the end of the lactation is frequently utilized to prevent and/or cure mastitis during the dry period.

There is abundant literature estimating the costs of the diseases listed above, using partial budgeting, simulation models, or dynamic programming [9–12]. The impact of each disease affecting lactating cows is often estimated separately for the various cost components of reduced milk production, discarded milk during treatment, extended days open until pregnant again, culling increase, death loss, veterinary cost, and labor cost [9–14]. Past evaluations of disease impacts were typically made under a business as usual assumption, i.e., antimicrobials are used to mitigate the impacts of diseases. The impacts and associated costs of diseases in a situation where no AMT is implemented remain surprisingly poorly studied, but such information is essential in order to evaluate the consequences of policy options aimed at reducing AMU in dairy production.

Using a stochastic simulation model calibrated with data available from the literature, our objective was to evaluate the cost impacts of a decrease in AMU in dairy herds, under modern dairy production conditions. We compared several hypothetical scenarios, corresponding to policies potentially implemented, to a baseline scenario mimicking the current field practices of AMU.

## Material and methods

A dairy farm simulation model constructed in Microsoft Excel (Microsoft, Redmond, WA) was used to evaluate the economic impact of constraints regarding AMU in cows producing milk. The basic model was built on biological and economic parameters of the dairy system, and estimated the net costs and benefits within a partial budgeting framework.

### Scenario definitions

#### Baseline scenario

The Baseline scenario corresponds to the current situation of AMU in a dairy. In the Baseline scenario, AMT are supervised by veterinarians, but no specific measure restricting their use is implemented. Nine ID were considered in our model: grade 1–2 clinical mastitis (CM_1-2_) (milk secretions ± mammary gland altered), grade 3 clinical mastitis (CM_3_) (milk secretions, mammary gland and general state altered), subclinical mastitis (SCM), retained placenta (RP), acute metritis (ACUTE-MET), endometritis (ENDOMET), digital dermatitis (DD-LAM), foot rot (FR-LAM), and respiratory disease (RESP). We assumed that for each ID, a non-specific AMT was implemented without restriction.

#### Prohibition scenario

Under the Prohibition scenario, AMU is not allowed in dairy production. We assumed that no substitution treatment was implemented.

#### Substitution scenario

Under the Substitution scenario, a substitution treatment is implemented. When possible, the substitution treatment corresponds to alternative protocols already tested in the field with available data of treatment effectiveness. If no substitution treatment was found, we assumed that under this scenario, the ID remained untreated. Alternative protocols were set for CM_1-2_, ENDOMET, LAM-DD and drying-off.

#### Cost increase scenario

Under the Cost Increase scenario, we increased the cost of AMT. The increase in cost might reflect a tax on AMT. We assumed that the cost increase affected all AMT classes proportionally, and that no substitutions between scenarios were made by farmers in this simulation in response to any cost increase. A cost increase could encourage a partial reduction in AMU, but the extent of any reduction was beyond the scope of this research.

#### Withdrawal period increase scenario

Under this scenario, we extended the milk withdrawal period from the current requirement of 0 to 3 days, to an increase of 5 to 15 days after treatment. The withdrawal period corresponds to a period following AMT, during which the milk collected from a treated cow potentially contains residues of drugs, and therefore is not authorized to be commercialized. An increase in the withdrawal period will indirectly increase the total costs of treatments, because more milk will be discarded from commercialization. The amount of milk discarded was the only component that was estimated to vary under this scenario, compared to the baseline scenario.

### Diseases prevalences

In each scenario, we modeled a generic herd of 1000 lactating cows with an assumed lactation length of 305 days, and a proportion of primiparous (first lactation) cows of 30%. Primiparous cows were assumed to produce 8,000 kg of milk per lactation, and multiparous (after the first lactation) cows produced 10,000 kg of milk per lactation [15].For each ID, we used an assumed estimate of the within herd prevalence (Pr). In the Baseline scenario, the default prevalences of CM_1-2_, CM_3_, SCM, ACUTE-MET, ENDOMET, RP, DD-LAM, FR-LAM, and RESP were set at 20%, 2.5%, 20%, 10%, 20%, 7%, 28%, 5%, and 3%, respectively, in accordance with the mean prevalences reported in several studies [6,16–29]. In addition, we determined two additional sets of values for disease prevalence, one representing a herd with a low level of disease, and a second an average herd with a high level of disease (Table 1).

**Table 1:** Prevalence estimates of diseases for model parametrization.

	Low Prevalence (%)	Mean Prevalence (%)	High prevalence (%)
**CM grade 1–2**	10	25	30
**CM grade 3**	1	2.5	5
**SCM**	5	20	28
**ACUTE-MET**	2	10	15
**ENDOMET**	10	20	30
**RP**	2	7	10
**LAM-DD**	10	28	38
**LAM-FR**	2	5	8
**RESP**	1	3	5

We estimated separately the cost components for both primiparous and multiparous cows, considering the potential discrepancies in the production performance and health impacts between these two subpopulations. However, when the estimates were not differentiated in the literature, we assumed that the estimates provided were valid for both primiparous and multiparous cows.

### Treatment practices

Antimicrobials are used in milk production to treat disease occurring during the lactation and to prevent or treat mastitis in the dry cow period. These two uses are discussed below with details of current practices, and then changes that would be required to implement alternative practices. Then, specific cost components for the various scenarios are presented.

#### Treatments during lactation

Almost all the treatments of the ID we studied in this paper fulfill a therapeutic curative objective: the occurrence of disease triggers the treatment, and the response to the treatment can be measured following the treatment period. In the baseline scenario, we assumed that the rates of AMT were 100%, 100%, 70%, 20%, 90%, 100%, 100%, and 100% for CM_1-2_, CM_3_, ACUTE-MET, ENDOMET, RP, DD-LAM, FR-LAM, and RESP, respectively (Francis Welcome, Cornell University, Personal communication). We assumed similar rates with alternative treatments in the substitution scenario. No treatment was administered in the Prohibition scenario. We did not consider the possibility that a cow would contract more than one disease, or the same disease multiple times in a lactation.

Treatment of SCM during lactation is not frequent. We assumed that only 10% of SCM cases were treated during lactation. In addition, we considered that each cow was treated during the dry-off period, regardless of the presence of elevated somatic cell counts in the milk at the end of the lactation (see below).

#### Dry cow therapy

Antimicrobial use for the treatment and prevention of mastitis at drying-off is likely to be the major source of antimicrobial consumption in dairy production, since a majority of farms implement a blanket dry cow therapy, consisting of treating each animal with intramammary antibiotics at drying-off [7,8]. For cows that do not exhibit signs of mastitis, the objective of the treatment is to prevent them from acquiring an infection during the dry period. For cows presenting mastitis, the objective is to cure the infection, so that the cow can start the following lactation free of pathogens and to prevent potential udder damage, both leading to a decrease in milk production. In the Baseline scenario, we assumed that 100% of the cows of the herd were given AMT at drying-off. The impact of the treatment is measured in the following lactation, considering two indicators: the percentage of new infections related to the preventive efficacy of the treatment, and the percentage of curative efficacy. In the Baseline scenario, we assumed that 20% of the cows exhibited SCM before drying-off. When AMT was modeled the preventive and curative efficacy rates were estimated at 80% and 75%, respectively (Table 2). Under these conditions, the prevalence of clinical and subclinical mastitis remained constant between lactations, and therefore the same values were used in the model in Years 1 and 2.

**Table 2:** Preventive and curative efficacy of the different drying-off antimicrobial treatments, and prevalence estimates of subclinical mastitis for multiparous cows in year 1 and 2 for model parametrization. BDCT: blanket dry cow therapy; DCT: dry cow therapy; TS: teat sealant.

Scenario	Intervention	Preventive efficacy (%)	Curative efficacy (%)	Prevalence (%)	
				Year 1	Year 2
**Baseline**	BDCT	80	75	20	20
**Prohibition**	No DCT	67	42	20	38
**Substitution**	TS	80	42	20	28

The impact of AMT prohibition at drying-off in Y1 will affect the prevalence of mastitis in Y2. In the Prohibition scenario, we assumed similarly that 20% of the cows were SCM in Y1 at drying-off. However, given that no AMT are used at drying-off, the number of multiparous cows with mastitis increases in Y2. This increased number of mastitis cases is associated with a decrease in the preventive and curative efficacy rates, compared to the Baseline scenario. The preventive and curative efficacy rates were calculated by adjusting the estimates retained in the Baseline scenario with relative risks (RR) found in the literature, according to the following formula: RR = incidence in treated group/incidence in non-treated group, for preventive efficacy, and RR = cure rate in treated group/cure rate in non-treated group, for curative efficacy [30,31]. The corrected values for preventive and curative efficacy rates were estimated at 67% and 42%, respectively (Table 2). Following this step, we calculated the number of infected cows in Y2 after drying-off; prevalence of SCM in Y2 was 38%.

An assumption of our model was that the percentage of infected cows before drying-off was constant (20%). Therefore, we adjusted the relative risk of being culled or dying in Y2 accordingly. Similar reasoning was followed to calculate the mastitis prevalence for multiparous cows in Y2 in the Substitution scenario. As suggested by the results of a meta-analysis, we assumed that the preventive efficacy treatments available at drying-off, such as teat sealants, were as good as antimicrobial therapy [32]. Robust data are missing concerning the curative efficacy of alternative treatments during the dry period [33]. We hypothesized that no treatment providing a higher efficacy than spontaneous cure was available. Therefore, the corrected values for preventive and curative efficacy rates were estimated at 80% and 42%, respectively, in the Substitution scenario (Table 2). Prevalence of SCM in year 2 was 28%.

### Evaluation of the impacts of ID

The impact of each disease was estimated for each of the different cost components: reduced milk production over a lactation (kg), discarded milk (kg), extended days open (d), anticipated culling (A-CULL) (RR), increased mortality (I-DEATH) (RR), veterinary and treatments costs ($), and labor costs ($). For reduced milk production, discarded milk and extended days open, the estimates were directly extracted from available literature and are shown in Table 3.

**Table 3:** Default parameters for estimating the impacts per cost component per disease.

		Primiparous									Multiparous								
		CM grade 1–2	CM grade 3	SCM	ACUTE-MET	ENDOMET	RP	LAM-DD	LAM-FR	RESP	CM grade 1–2	CM grade 3	SCM	ACUTE-MET	ENDOMET	RP	LAM-DD	LAM-FR	RESP
**Reduced milk** **production (kg)**	**Baseline scenario**	130	220	150	120	0	0	250	250	120	200	300	300	120	0	0	250	250	120
	**Prohibition scenario**	230	230	250	250	0	0	500	500	250	450	450	400	250	0	0	500	500	250
	**Substitution scenario**	180	180	250	180	0	0	250	400	180	350	400	400	180	0	0	250	450	180
**Extended days open** **(d)**	**Baseline scenario**	0.4	0.4	0	18	18	11	12	12	0	0.4	0.4	0	18	18	11	12	12	0
	**Prohibition scenario**	15	15	0	68	68	58	30	30	30	15	15	0	68	68	58	30	30	30
	**Substitution scenario**	8	8	0	40	40	11	12	30	30	8	8	0	40	40	11	12	30	30
**RR anticipated** **culling**	**Baseline scenario**	2.2	2.2	1.3	1.5	1.5	2	1.5	1.5	1.2	2.5	2.5	1.3	1.4	1.4	1.4	1.5	1.5	1.2
	**Prohibition scenario**	4	4	3	2.5	2.5	2	2	4	4	4	4	1.7	2.5	2.5	2	2	4	4
	**Substitution scenario**	3	3	1.5	2	2	2	1.5	3	3	3	3	1.5	2	2	2	1.5	3	3
**RR increased** **death**	**Baseline scenario**	1	8	1	5	1	1	1	1	5	1	8	1	5	1	1	1	1	5
	**Prohibition scenario**	1	12	1	6	1	1	1	1	6	1	12	1	6	1	1	1	1	6
	**Subsitution scenario**	1	10	1	6	1	1	1	1	5	1	10	1	6	1	1	1	1	5
**Number of anticipated** **culling**	**Baseline scenario**	8.1	1.2	2.4	2.0	3.8	2.7	5.2	1.0	0.3	22.6	3.5	5.5	3.8	7.3	2.7	12.0	2.4	0.6
	**Prohibition scenario**	15.8	2.9	12.0	5.5	9.7	2.7	9.2	5.5	3.5	36.8	6.8	12.0	12.8	22.6	6.4	21.4	12.8	8.1
	**Substitution scenario**	12.0	2.0	3.8	3.8	7.0	2.7	5.2	3.8	2.4	28.0	4.7	8.9	8.9	16.3	6.4	12.0	8.9	5.5
**Number of** **death**	**Baseline scenario**	0.0	2.2	0.0	4.3	0.0	0.0	0.0	0.0	1.6	0.0	5.2	0.0	10.0	0.0	0.0	0.0	0.0	3.8
	**Prohibition scenario**	0.0	3.2	0.0	5.0	0.0	0.0	0.0	0.0	2.0	0.0	7.5	0.0	11.7	0.0	0.0	0.0	0.0	4.6
	**Substitution scenario**	0.0	2.8	0.0	5.0	0.0	0.0	0.0	0.0	1.6	0.0	6.4	0.0	11.7	0.0	0.0	0.0	0.0	3.8

CM1-2: grade 1–2 clinical mastitis; CM3: grade 3 clinical mastitis; SCM: subclinical mastitis; RP: retained placenta; ACUTE-MET: acute metritis; ENDOMET: endometritis; DD-LAM: digital dermatitis; FR-LAM: foot rot; RESP: respiratory disease; RR: relative risk

For the Baseline scenario, estimates of impacts provided by previous research were generally extracted from studies in which AMT were used to manage infectious diseases. Therefore, we used an average value from the range from the literature for each cost impact component, assuming that AMT were performed to achieve these values. For the Prohibition scenario, the values of each component impact were set at the highest disease impact estimate found in the literature, under the assumption that no control measure would produce these higher disease impacts. For the Substitution scenario_,_ the values of each component impact were set at an intermediate level between the average and highest disease impacts. We fixed these values considering the presence of an available substitution treatment for which data of treatment effectiveness were published. If no treatment was available, we fixed the same values as for the Prohibition scenario.

For A-CULL and I-DEATH, we estimated the values of Relative Risk (RR) of culling and death for each ID and for each of the cost components, and inferred a possible range of variation around the estimates (Table 3). The population attributable fractions were then calculated, as described in the following formula [34].

$${AF}_{p}=\frac{p(E+)(RR-1)}{p(E+)(RR-1)+1}$$

where:

AF_p_: population attributable fraction

p(E+): prevalence of ID

RR: relative risk of culling or death associated with ID

Assuming a cull rate in the herd of 28% and a death rate of 5% [35,36], we then calculated the number of attributable cows that were culled and died for each disease. For simplification purposes, diseases are modeled as only occurring once and are independent from each other. Since in reality some cows in the herd are culled while they experience more than one ID simultaneously, and that some are culled without any disease, we adjusted the number of culled animals having disease by considering that at least 50% of cows were culled without disease, as reported by Pinedo et al. [37]. The number of anticipated culling and death in each scenario are presented in Table 3. An assumption of our model was to keep the herd at a steady state; therefore, the variations in the RR and the prevalence of disease between the different scenarios do not affect directly the total number of cows culled, but change the repartition of the culling motives across the total number of culling.

### Costs calculation

#### Cost estimates

First, an average cost was estimated for each cost component, according to recent published data (Table 4). For the estimation of milk losses and discarded milk costs, milk price was based on the U.S. average price from the previous five years (2013–2017) at $0.42/kg [38]. The cost of an extended day open was estimated based on the average reported in the literature and set at $4/day [38–42].

**Table 4:** Input and output prices used in stochastic simulation. Milk price, Total Mixed Ration price and meat price were assumed to be normally distributed; for the other parameters, a triangular distribution was used.

Component	Mean	Standard deviation	Mode	Min-Max	Reference
**Milk price ($/kg)**	0.42	0.07			USDA, 2017
**Non saleable milk ($/kg)**	0.33	0.1			Rollin et al., 2015
**Total Mixed Ration ($/kg)**	0.195	0.04			Wisconsin University, 2017
**Extended days open cost ($/day)**			4	3.6–4.4	Groenendal et al., 2004 De Vries et al., 2006 Liang, 2013
**Meat price ($/kg)**	1.96	0.2			USDA, 2017
**Slaughter body weight primiparous (kg)**			500	450–550	Rollin et al., 2015
**Slaughter body weight multiparous (kg)**			750	700–800	Rollin et al., 2015
**Replacement primiparous ($)**			2094	1885–2303	USDA, 2017; Wisconsin University, 2017
**Replacement multiparous ($)**			1761	1761–1937	USDA, 2017; Wisconsin University, 2017

The cost of anticipated culling was calculated as previously described by Rollin et al. [13], i.e., the estimate of attributable anticipated culling associated with the ID was multiplied by the difference of the value of a healthy animal minus the value of a culled animal. The cost of increased mortality was calculated as the estimate of attributable mortality associated with the ID_,_ multiplied by the value of a healthy animal.

Estimates of veterinary, treatment, and labor costs were derived from the results of a survey described by Liang et al. [40] (Table 5). The costs of non-specific AMT were set for each disease, according to values provided by Cornell University Quality Milk Production Services (Table 5).

**Table 5:** Default parameters for estimating the costs of treatments and costs of discarded milk. BS: Baseline Scenario; PS: Prohibition scenario; SS: Substitution scenario; CM1-2: grade 1–2 clinical mastitis; CM3: grade 3 clinical mastitis; SCM: subclinical mastitis; RP: retained placenta; ACUTE-MET: acute metritis; ENDOMET: endometritis; DD-LAM: digital dermatitis; FR-LAM: foot rot; RESP: respiratory disease.

Disease	Veterinary costs ($)			Labor costs ($)			Non antimicrobial treatment costs ($)			Antimicrobial treatment costs ($)			Days of treatment (days)			Withdrawal period (days)		
	BS	PS	SS	BS	PS	SS	BS	PS	SS	BS	PS	SS	BS	PS	SS	BS	PS	SS
**CM grade 1–2**	19.16	0	19.16	11.58	0	11.58	0	0	20	20	0	0	3	0	3	3	0	0
**CM grade 3**	19.16	0	19.16	11.58	0	11.58	80	0	100	20	0	0	3	0	0	3	0	0
**SCM**	0	0	0	11.58	0	11.58	0	0	0	30	0	0	6	0	0	1.5	0	0
**ACUTE-MET**	21.81	0	21.81	9.74	0	9.74	80	0	80	40	0	0	2	0	0	0	0	0
**ENDOMET**	21.81	0	21.81	9.74	0	9.74	0	0	20	20	0	0	2	0	2	0	0	0
**RP**	17.61	0	17.61	11.86	0	11.86	0	0	20	30	0	0	2	0	0	3	0	0
**LAM-DD**	36.57	0	36.57	13.1	0	13.1	0	0	10	10	0	0	2	0	2	0	0	0
**LAM-FR**	36.57	0	36.57	13.1	0	13.1	0	0	0	40	0	0	2	0	0	0	0	0
**RESP**	21.81	0	21.81		9.74	0	9.74		80	0	80		40	0	0		2	0	0		0	0	0

The total cost was estimated for each disease, by multiplying the costs described above with the estimates for each component, and summed in each scenario.

#### Cost and revenue adjustments

We calculated the feed costs saved in the case of decreased milk production. We considered that 1 kg of dry matter intake (DMI) was consumed to produce 2 kg more milk over a maintenance diet, and reciprocally, that a decrease of 2 kg in milk production led to a decrease of 1 kg of DMI [43]. The price of a kg of DMI was set at $0.195 [38]. The amount of discarded milk is the milk produced during the period of AMT and the withdrawal period following the treatment, when milk cannot enter the food chain. Current on farm practice consists of using the discarded milk to feed non-weaned calves. The value of a kg of discarded milk was set at $0.33 (saleable milk price ($/kg) * 0.785), based on the methodology published by Rollin et al. [13]. The value of the adjustments was then subtracted from the crude cost to obtain the net cost for each scenario.

#### Comparison of scenarios

We computed the cost of each scenario over a two-year period, which takes into account the impact of one drying-off period on the succeeding second year lactation. Then, cost of each scenario tested was compared to the baseline; a cost difference was measured by subtracting the net cost for the Baseline scenario from the net cost of each scenario tested (Eq 1 and 2). Thus:

*Cost difference in Prohibition scenario = net costs Prohibition scenario–net costs Baseline scenario (1)*, and

*Cost difference in Substitution scenario = net costs Substitution scenario–net costs Baseline scenario (2)*.

### Sensitivity analysis

The @Risk (Palisade, Ithaca, NY) add-in was used to perform sensitivity analysis of the influence of stochastic and deterministic input parameters on outcome values. Input and output prices were modeled stochastically (Table 4). To take into account differences in the levels of disease prevalence between herds, we also determined 2 additional levels of prevalence (low and high) to the mean level of prevalence (Table 1). This permitted an assessment among farms of various disease incidences. In the Cost increase scenario, we increased incrementally from 1.5 to 5 fold the price of AMT used in the Baseline scenario. In the Withdrawal period increase scenario, we increased incrementally by a step of 5 days the length of the milk withdrawal period.

## Results

### Cost difference under the Prohibition and Substitution scenarios

**Fig 1:**
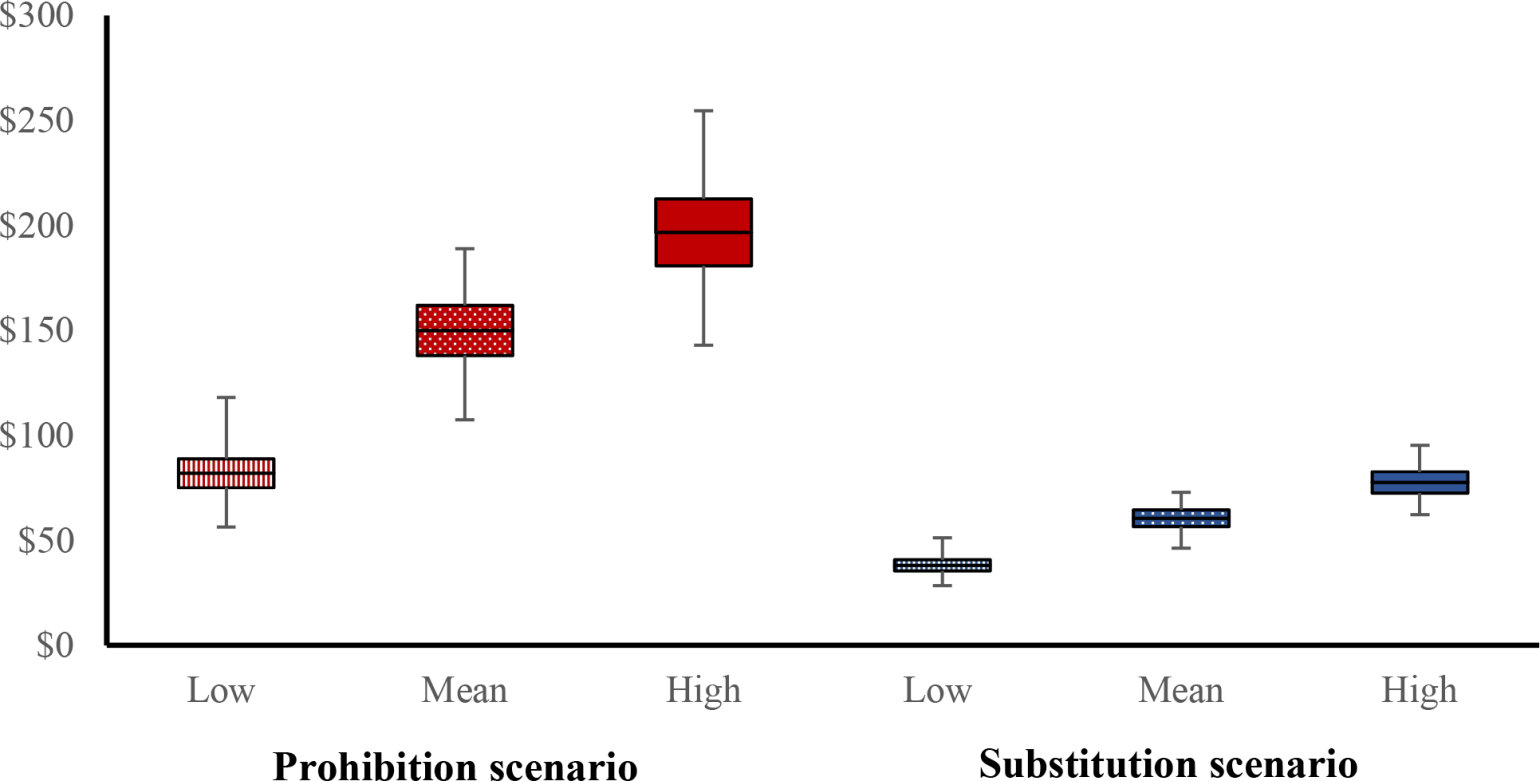
Total net costs per cow per year for the Prohibition scenario and Substitution scenario, as a function of the level of disease prevalence (low, mean, high). The boxes represent mean ± SD, and the bars extend from minimum to maximum values. The simulation was run with 5000 iterations.

The estimated cost differences of mitigating AMU under each scenario are presented in Table 6 and displayed in Fig 1. With a mean prevalence, the average increase in costs over current AMU were $150±12 Sd per cow per year for the Prohibition scenario and $61±4 for the Substitution scenario. In the Prohibition scenario_,_ the model predicted that the minimum and maximum increased costs associated with AMU prohibition were $107 and $189 per cow. In the Substitution scenario_,_ the model predicted that the minimum and maximum increased costs associated with AMU prohibition were $46 and $73 per cow. In each scenario, the cost difference increased with prevalence.

**Table 6:** Average, standard deviation, minimum and maximum, per cow per year values of cost increases, in the Prohibition and Substitution scenarios, by the level of prevalence.

	Prohibition scenario			Substitution scenario		
Prevalence level	Low	Mean	High	Low	Mean	High
**Average** **cost increase ($)**	82	150	197	38	61	78
**SD**	7	12	16	3	4	5
**Min**	56	107	143	28	46	62
**Max**	** **	118	189	255	51	73	95

The results of the sensitivity analysis indicated that the three stochastic variables most influencing cost differences were respectively, cow replacement prices, cow slaughter price, and milk price.

**Fig 2:**
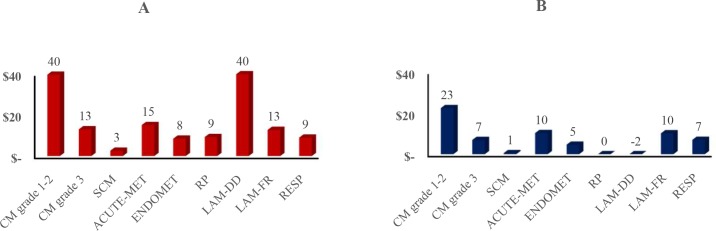
**Repartition of the cost difference per cow per year in the Prohibition scenario (2A) and the Substitution scenario (2B), per type of infectious disease.** CM1-2: grade 1–2 clinical mastitis; CM3: grade 3 clinical mastitis; SCM: subclinical mastitis; RP: retained placenta; ACUTE-MET: acute metritis; ENDOMET: endometritis; DD-LAM: digital dermatitis; FR-LAM: foot rot; RESP: respiratory disease.

The breakdown of the net costs per infectious disease shows that the quantitative contribution to the total costs varied significantly among diseases (**Fig 2A and 2B**). Grade 1 and 2 clinical Mastitis (CM_1-2_) accounted for approximately 28% and 38% of the total costs and represented the largest costs in both scenarios. The second largest cost was LAM-DD in the Prohibition scenario, and ACUTE-MET in the Substitution scenario, accounting for 25% and 15% of total disease cost. The costs of mastitis remained constant between Y1 and Y2 in the Baseline scenario. Because of the increased prevalence of mastitis in the Prohibition scenario and the Substitution scenario, the cost of mastitis increased by 2.26 fold in the Prohibition scenario and by 1.8 fold in the Substitution scenario.

**Fig 3:**
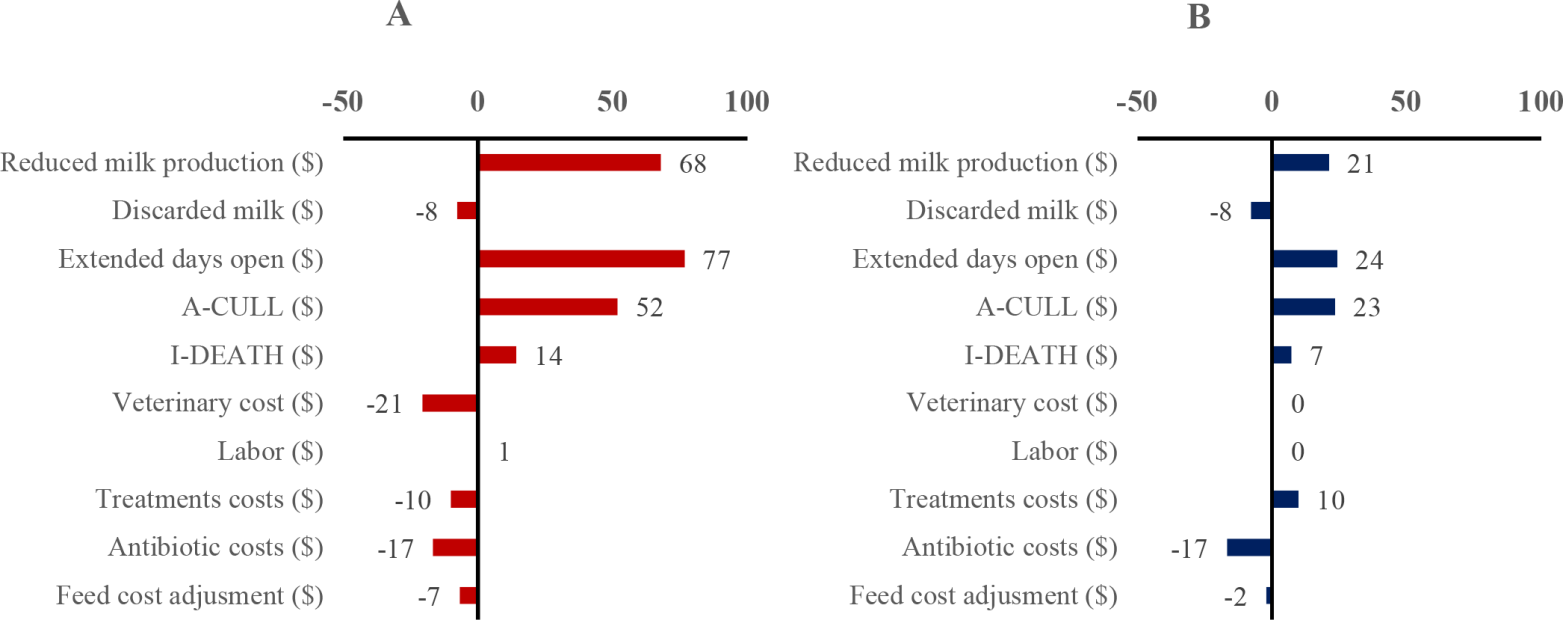
**Repartition of the cost difference per cow per year in the Prohibition scenario (3A) and the Substitution scenario (3B), per impact cost component.** A-CULL: anticipated culling; I-DEATH: increased mortality.

The breakdown of costs by cost components shows that reduced milk production, increase in extended days open, and increase in anticipated culling were the 3 largest costs associated with non-usage of AMT (**Fig 3A and 3B**). With a mean prevalence, the cost increases for reduced milk production, extended days open and A-CULL were $68, $77 and $52, respectively, in the Prohibition scenario. In the Substitution scenario, the average cost differences for reduced milk production, extended days open and A-CULL were $21, $24 and $23, respectively.

#### Per case costs

The estimates of costs per case in each scenario are depicted in Table 7.

**Table 7:** Per case costs of diseases for primiparous and multiparous cows, in the Baseline, Prohibition and Substitution scenarios. CM1-2: grade 1–2 clinical mastitis; CM3: grade 3 clinical mastitis; SCM: subclinical mastitis; RP: retained placenta; ACUTE-MET: acute metritis; ENDOMET: endometritis; DD-LAM: digital dermatitis; FR-LAM: foot rot; RESP: respiratory disease.

	Baseline scenario Per case costs ($)		Prohibition scenario Per case costs ($)		Substitution scenario Per case costs ($)	
Disease	Primiparous	Multiparous	Primiparous	Multiparous	Primiparous	Multiparous
**CM grade 1–2**	296	264	435	363	362	308
**CM grade 3**	1,066	926	1,468	1,172	1,278	1,081
**SCM**	210	264	320	213	181	204
**ACUTE-MET**	658	568	921	751	825	690
**ENDOMET**	206	165	446	345	330	257
**RP**	280	179	377	295	226	144
**LAM-DD**	292	258	454	386	275	237
**LAM-FR**	329	291	714	485	596	458
**RESP**	621	549	1,081	767	949	724

In each scenario, grade 3 clinical mastitis, acute metritis, and respiratory diseases were the 3 costliest diseases per case. For all diseases except subclinical mastitis, the costs were slightly higher for primiparous than for multiparous cows.

### Impact of antimicrobial treatment prices

The results of an incremental increase in initial AMT costs by various factors are depicted in Table 8.

**Table 8:** Sensitivity analysis of an increase of initial antimicrobial price on the net costs of diseases. AMT: antimicrobial treatment.

	Baseline Scenario	Cost increase scenario					Substitution scenario	Prohibition scenario
AMT price	Initial price x 1	x 1.5	x 2	x 3	x 4	x 5	-	-
**AMT costs ($)**	17	25	33	50	66	83	0	0
**Net costs ($)**	369	377	386	402	419	435	430	519

With a 5-fold increase in the initial AMT price, the net costs associated with diseases increased from $369 to $435. With a 5-fold increase in AMT price, net costs of the treatments were higher than those observed when the Substitution alternative treatment was implemented.

### Length of milk withdrawal period

The results of an increase from the current milk withdrawal period up to 15 additional days over current withholding period by antibiotic as summarized in the appendix table are depicted in Table 9.

**Table 9:** Sensitivity analysis of a 5-day to 15-day increase of initial withdrawal period on the net costs of diseases. WP: withdrawal period.

	Baseline Scenario	Withdrawal period increase scenario			Substitution scenario	Prohibition scenario
	initial WP	WP+5	WP+10	WP+15	-	-
**Discarded milk costs ($)**	8	18	29	40	0	0
**Net costs ($)**	369	415	426	437	430	519

The net costs associated with diseases when AMT was used increased from $369 to $437 with a 15-day increase in the withdrawal period. In such situations, net costs of the treatments were higher than those observed in the Substitution alternative treatment.

## Discussion

Our study modeled the impacts of potential policies restricting the use of antimicrobials in dairy production. We hypothesized that farmers would either need to cease the use of antimicrobials or find alternative treatments, leading us to specify and estimate both Prohibition and Substitution scenarios. In addition, we assessed the impacts of hypothetical increases in AMT prices, and lengths of milk withdrawal periods, in scenarios of AMT. After scenario costs were estimated, these costs were differenced from the cost of the Baseline scenario of AMU, which is the current practice.

The average cost differences per cow per year were $150±12 and $61±4 in the Prohibition scenario and the Substitution scenario respectively, under mean disease prevalence conditions. The Prohibition scenario corresponds to a situation in which any disease affecting an animal remains untreated; therefore, the costs of diseases in the Prohibition scenario represent an upper estimate. The determination of costs in the Prohibition scenario was a necessary step because in the field, some diseases remain untreated, and also because alternatives to AMT do not always exist for some infectious diseases studied in our model. With any restriction on AMU, farmers will probably implement available alternative treatments, corresponding to the Substitution scenario, for two reasons. First, as available alternatives exist, they should be utilized by the farmers as a second best strategy to limit the costs of disease. Second, animal welfare concerns mandates care of a diseased animal, and in case of prohibition, it is unlikely that such an animal would remain untreated. Nonetheless, it is likely that the number of animals culled would increase, because of a potential decrease in the efficacy of the substitution treatment. To model this possible occurrence, we increased the cull rates in the Prohibition and the Substitution scenarios. Therefore, the costs estimated in the Substitution scenario fit with a situation of AMT restriction. Considering the Substitution scenario, we estimated the minimum and maximum yearly additional costs per lactating cow at $46 and $73. These values represent 0.7 and 1.2% of the yearly total production cost for a lactating cow evaluated in 2016 at $5754 [39].

The main components of total net costs stemmed mainly from reduced milk production and costs associated with fertility and reproductive disorders (extended days open and metritis). Our results are in agreement with other studies investigating the costs of several dairy cows’ diseases [11,12,17,40,44–51]. A first step of our simulation was to determine the per case costs for each disease. Our estimates of these impacts were in the same range of results previously published. As an example, average per case cost of CM_1-2_ for a multiparous cow was $264 in our study, with recent estimates providing values spanning from approximately $100 to $450 [12,13,40,52].

The initial input values regarding the impacts of the different infectious diseases were extracted from available literature. As our purpose was to simulate a generic herd situation, we first targeted available data in meta-analysis and systematic reviews [17,20,22,23,30–32,45,47,52–57]. If needed, supplementary estimates were added to determine the average impact and an associated range. The costs of production inputs and outputs were estimated on the basis of years 2013 to 2017 market prices. As agricultural markets can experience high volatility, we modeled these variables stochastically. We conducted a sensitivity analysis of our outputs to a set of input values, to investigate the relative importance of the input values. Cow replacement price, slaughter price and milk price were found to be the major factors explaining the variability in total net costs. Regardless of the influence of the parameters, the results of the sensitivity analysis showed that there was a 90% likelihood that the total net costs ranged between $57 and $70 in the Substitution scenario.

We evaluated the costs of AMU prohibition simulating a herd with average disease prevalences, based on data reported in the literature. To assess the sensitivity of the outcome to a decrease or increase of prevalence, we also simulated low and high prevalence situations. As expected, the cost difference increased with prevalence in both Prohibition and Substitution scenarios. Interestingly, the cost difference increased at a higher rate in the Prohibition scenario than in the Substitution scenario as the prevalence increased, showing that alternative treatments allowed some cost mitigation of AMU prohibition, regardless of the initial prevalence.

Our approach simulated two scenarios in which AMT was no longer available (the Prohibition and Substitution scenarios). These scenarios mimic strong constraints imposed on farmers, requiring immediate changes in the farming system. We also evaluated the impact of an incremental increase in AMT prices. Such an increase mimics a potential tax on AMU. Recent studies have mentioned implementation of taxes on antimicrobials in animal agriculture as a way to mitigate the risk of AMR [5,58]. Under the conditions of our model, we found that a 5-fold increase in AMT price was necessary to reach similar total net costs as observed in the Substitution scenario, showing that an increase lower than 5-fold was not sufficient to encourage the use of alternative non AMT to cure infectious diseases. These results suggest that an extremely significant cost increase, i.e., a tax, would probably be required to encourage farmers to cease the use of AMT and move to the alternative treatment. In actuality, what might occur on farms with an AMT price increase is that only some diseases would be treated, depending upon the effectiveness of AMT on each disease and the per case costs of each disease. According to the differences in our per case costs estimates between diseases, the expected benefits of treatment will probably lead farmers to prioritize AMT of diseases for which the impacts are highest. Although beyond the scope of our study, the evaluation of farmers’ decision-making behavior facing regulations of AMU would be necessary to determine their behavior change.

We also evaluated the impact of an incremental increase in the length of the withdrawal period. The rationale of this evaluation was to evaluate the effect of a potential regulation aiming to set a withdrawal period associated with AMU to ensure food safety, and to curb AMR. Currently, the lengths of withdrawal periods are determined to guarantee a concentration of antimicrobial residues below a regulatory threshold in animal products entering the food chain. A recent study showed that for chlortetracycline distributed orally to cattle, the withdrawal period was not sufficient to allow a return to a baseline level of the proportion of resistant bacteria selected by AMT [59]. These authors determined that an increase of 15 to 36 days of the withdrawal period was required to limit the risk of transmission via the food chain of resistant bacteria selected during AMT. Without information regarding AMR in our study, we simply calculated the length of withdrawal period that should lead to total net costs above those estimated in the Substitution scenario. We evaluated that this initial withdrawal period increase would be 15 days. In practice, such an increase results in milk withheld during approximately 5% of the lactation time, a significant loss in revenue with continued cost to the farm.

One limitation of our model is that the dynamics aspects of diseases, such as transmission between animals, and potential adjustments made by the farmers, were not considered. Agent based models may be helpful to capture the dynamics of disease transmission within the herd and to test several treatment strategies potentially implemented by farmers, such as selective AMT targeting specified categories of animals [60,61]. The interest of such modeling also lies in the ability to trace the impact of concurrent disease cases and their severity. As an example, the prevalence of subclinical ketosis has been shown to impact AMU [62]. In addition, depending on the incidence and contagiousness of the disease, the costs associated with AMU prohibition may be lower or higher than the average costs estimated in our study. The treatment of subclinical mastitis represents a good example of such variation because depending upon the transmission rate of the specific pathogen species, treatment during lactation may or may not be beneficial [63]. AMU prohibition might lead to a continuous degradation of the herd health status, associated with an incremental increase in the number of cases over time.

For our purpose, we considered that contagiousness of bacterial diseases studied in our model was sufficiently low to be neglected, and that the impact of a treatment failure for an animal afflicted with one disease did not affect the herd prevalence of the same disease. However, regarding the purpose of AMT during the dry period, aiming both to prevent and cure mastitis at the herd level, we simulated our scenarios over two years, in order to include the consequences of restrictions affecting antimicrobial dry cow therapy. Potential substitutions between preventive options such as vaccination and curative options were beyond the scope of our study. Still, the costs associated with AMU prohibition should encourage the use of preventive options and biosecurity, as long as they exhibit an economic benefit. A longer time frame might be considered to evaluate the relationship between restrictions regarding AMU, and the development of preventive measures in the herds.

In our model, the per case cost impacts were set with regards to previous published data. As mentioned, these data refer to empirical situations, in which AMT are part of the strategy of disease control. As tools of damage control, antimicrobials should be used only in situations in which their marginal costs are less than or equal to the expected marginal benefits. These marginal benefits depend widely on antimicrobial effectiveness. Interestingly, AMT effectiveness was not reported in the vast majority of research studies estimating the costs of disease. Further evaluation of AMT effectiveness on disease impacts might be useful, especially to assess the costs and benefits of specific treatment strategies.

In conclusion, our results suggest that under current U.S. dairy production conditions, restricting AMU might have a moderate economic impact at the herd level. However, policies aimed at changing farmers’ behavior regarding AMU should be carefully evaluated before being implemented, particularly given the relative current low costs of antimicrobials and the complexity of farmers’ decision making regarding animal health.
